# Trends in Chitosan as a Primary Biopolymer for Functional Films and Coatings Manufacture for Food and Natural Products

**DOI:** 10.3390/polym13050767

**Published:** 2021-03-01

**Authors:** Elsa Díaz-Montes, Roberto Castro-Muñoz

**Affiliations:** 1 Unidad Profesional Interdisciplinaria de Biotecnología, Instituto Politécnico Nacional, Av. Acueducto s/n Col. Barrio La Laguna Ticoman, Mexico City 07340, Mexico; elsadimo123@gmail.com; 2 Tecnologico de Monterrey, Campus Toluca. Av. Eduardo Monroy Cárdenas 2000 San Antonio Buenavista, Toluca de Lerdo 50110, Mexico; 3 Department of Process Engineering and Chemical Technology, Faculty of Chemistry, Gdansk University of Technology, 11/12 Narutowicza St., 80-233 Gdansk, Poland

**Keywords:** chitosan, edible films, edible coatings, quality food, conservation, shelf-life

## Abstract

Some of the current challenges faced by the food industry deal with the natural ripening process and the short shelf-life of fresh and minimally processed products. The loss of vitamins and minerals, lipid oxidation, enzymatic browning, and growth of microorganisms have been the main issues for many years within the innovation and improvement of food packaging, which seeks to preserve and protect the product until its consumption. Most of the conventional packaging are petroleum-derived plastics, which after product consumption becomes a major concern due to environmental damage provoked by their difficult degradation. In this sense, many researchers have shown interest in edible films and coatings, which represent an environmentally friendly alternative for food packaging. To date, chitosan (CS) is among the most common materials in the formulation of these biodegradable packaging together with polysaccharides, proteins, and lipids. The good film-forming and biological properties (i.e., antimicrobial, antifungal, and antiviral) of CS have fostered its usage in food packaging. Therefore, the goal of this paper is to collect and discuss the latest development works (over the last five years) aimed at using CS in the manufacture of edible films and coatings for food preservation. Particular attention has been devoted to relevant findings in the field, together with the novel preparation protocols of such biodegradable packaging. Finally, recent trends in new concepts of composite films and coatings are also addressed.

## 1. Introduction

**Figure 1:**
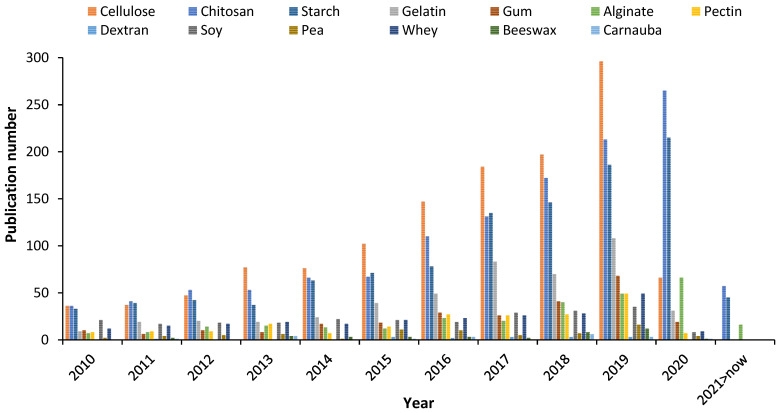
Publication number related to the fabrication of food packaging based on biopolymers (source Scopus, keywords: cellulose, chitosan, starch, gelatin, gum, alginate, pectin, dextran, soy, pea, whey, beeswax, and carnauba. Accessed: 22 February 2021).

Recently, it was estimated worldwide that more than 66 million tons of petroleum-based plastics were manufactured specifically for food packaging, including trays, cups, and bottles [1]. The manufacturing of synthetic packaging requires non-renewable natural resources that take many years to degrade, hence its use has been initiated to be banned [2]. To face the environmental pollution by conventional packaging, the packaging based on natural materials has recently received considerable attention due to the advantages over petroleum-based plastics [1], such as natural origin, no toxicity, recycling, biocompatibility, and biodegradability [3,4]. Biodegradable packaging based on renewable raw materials are indeed a viable alternative for preserving the environment [5]. Edible films and coatings arise from the need to reduce the consumption of petroleum-based plastics in food products’ packaging while allowing to maintain quality of foods and extend their shelf-life [6]. To date, several biopolymers have been utilized in the formulation of packaging including starch, gums, gelatin, pectin, and chitosan (CS) [7]. The latter is likely the most investigated biopolymer for biodegradable packaging fabrication, which is reflected in the growing number of publications in the field, as illustrated in Figure 1. Over the last decade, CS has been exponentially explored and even more studied than cellulose, which is the most abundant biopolymer in nature and appreciated for its differentiated polyfunctionality that allows to apply it as a packaging material [8]. The literature has shown the increasing research interest in CS for different concepts of food packaging. Thus, the aim of this review is to show the recent advances (over the last five years) in CS-based edible films and coatings, and their application in food systems, together with the possible interactions and benefits offered to the foods. Also, a brief background on the CS properties is introduced. To finalize, the role of CS in the preparation of functional and new concepts of composite films and coatings is reviewed.

## 2. Properties and Bioactivity of Chitosan

### 2.1. Properties of Chitosan

**Figure 2:**
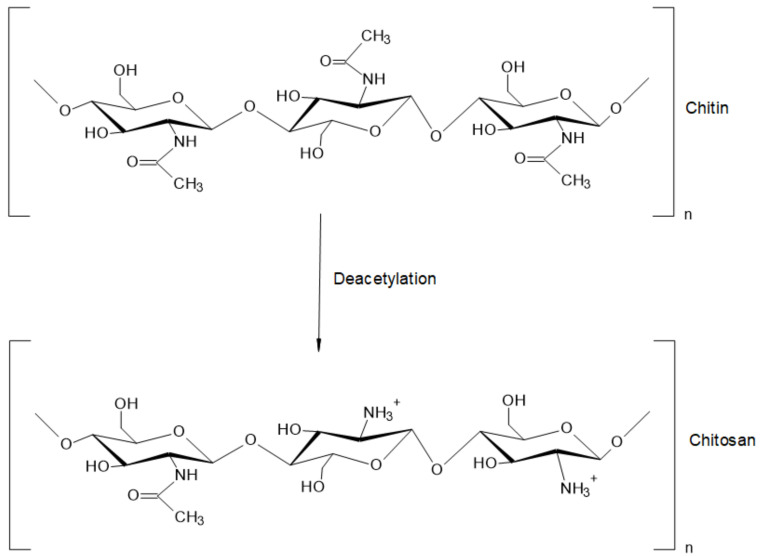
Structure of chitin and chitosan.

Chitosan (CS) is a natural biopolymer derived by deacetylation (N-acetyl-D-glucosamine to D-glucosamine unit) of chitin (see Figure 2), which has been studied for its effectiveness against bacterial, fungal, and viral pathogens [9]; in addition, CS has demonstrated minimal toxicity in mammals and humans [10]. CS owns a molecular weight (MW) between 10 and 1000 kDa [11], but the MW and degree of acetylation (DA) depend on the type of source [12]. In principle, the DA is defined as the proportion of N-acetyl-D-glucosamine units with respect to the total number of units [11,13]. CS can be obtained from several sources including sea animals (e.g., annelida, mollusca, coelenterate, and crustaceans), insects (e.g., scorpions, spiders, ants, cockroaches, and beetles), microorganisms (e.g., algae, yeast, fungi, ascomycetes, and spores), among others [14]. The different MW and DA of chitosan also influence its physicochemical properties, such as solubility, appearance, rheological properties, among others [11], directly impacting the bioactivity and toxicity of the polysaccharide [10].

CS is a viscous polysaccharide that forms more structured gels when its MW increases; furthermore, it is considered as a pseudoplastic material since its viscosity is a function of concentration, temperature, and rate of shear [11]. The solubility of CS varies depending on DA, and it is insoluble in neutral-alkaline pH but highly soluble in acid pH due to the proportion of protonated amino groups (-NH_2_) that are positively charged [15]. Depending on its DA, the CS-polyelectrolyte takes different behaviors in solution; for example, if DA is greater than 50%, the molecule maintains the hydrophobic characteristics of chitin, while for a DA between 20% and 50%, the molecule becomes less hydrophobic, and CS, having a DA less than 20%, is considered a highly hydrophilic cationic polyelectrolyte [16]. CS can form amorphous and complex three-dimensional structures due to its polycationic nature that allows it to interact electrostatically (by hydroxyl groups), or to form covalent bonds by -NH_2_ groups, with other molecules, such as metals, surfactants, proteins, and polyanions [14].

### 2.2. Bioactivity of Chitosan

**Figure 3:**
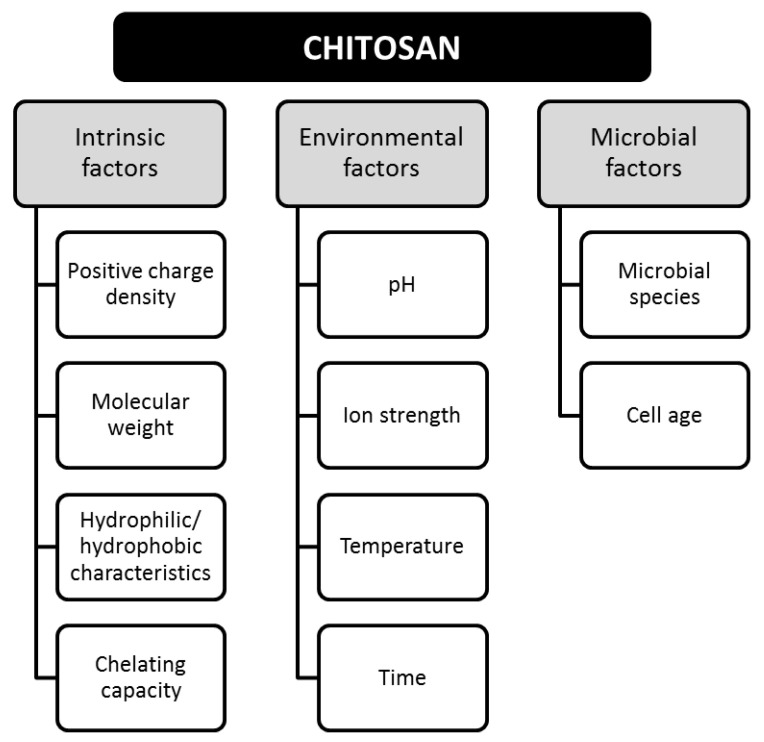
Factors involved in the bioactivity of chitosan. Adapted from Reference [16].

Bioactivity is defined as the ability of a material to chemically interact with compounds, microorganisms, or pathogens [17]. The bioactivity of CS has been related to the antimicrobial, antifungal, and antiviral properties, providing important contributions in different applications, such as additives, agro-food, pharmaceutical, and medical [18]. Bioactivity is determined by intrinsic, environmental, and microbial factors, as shown in Figure 3.

The intrinsic properties can be modified by varying the temperature and time of exposure when CS is in dispersion [16]. The antimicrobial activity is totally dependent on the pH of the medium and the solubility of CS [19]. For instance, a stronger inhibitory effect has been demonstrated at lower pH, such inhibitory effect decreases as the pH increases, and this is attributed to the neutralization of the -NH_2_ groups in the alkaline medium [16,20]. CS can also act as a chelator for metal ions (e.g., nickel, zinc, cobalt, iron, magnesium, and copper) [21], and some studies promote its addition to modify the ionic strength [16], however divalent ions have been shown to attenuate the inhibitory activity of CS [22]. Regarding the antimicrobial effect of CS, Table 1 lists some of the pathogens that have been inhibited by CS.

**Figure 4:**
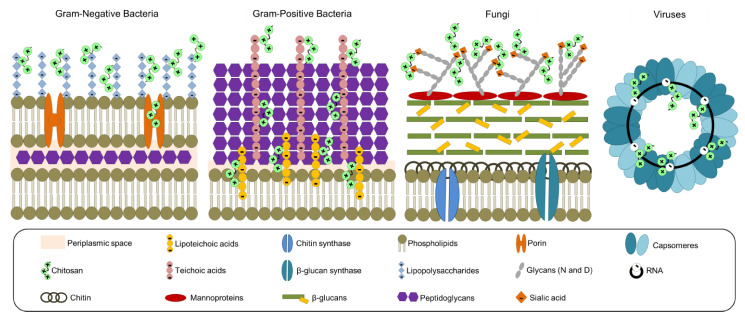
Proposed interactions and mechanism of action between chitosan and pathogens (Gram-positive and Gram-negative bacteria, fungi, and viruses). Adapted from References [24,25].

According to the insights documented by several authors, the antibacterial effect of CS on prokaryotic cells (e.g., Gram-negative and Gram-positive bacteria) depends on the electrostatic interactions between the biopolymer and bacterial cell wall components (e.g., teichoic and lipoteichoic acids, and lipopolysaccharides), altering stiffness and eventually entering into the cell [25]. The antifungal effect of CS on eukaryotic cells is thanks to the biopolymer cationic power acting on the residues of the mannoproteins (i.e., sialic acid) located on the surface; once inside the cells, CS can chelate metals (e.g., potassium, calcium, and sodium), denature proteins due to the effect of the net negative charge, or interact with DNA via nucleic acid phosphate groups [25]. The antiviral effect may be due to the interaction between CS and the RNA of viruses via phosphate groups [24]. To sum up, Figure 4 represents most of the reported mechanisms of action of CS toward pathogens.

Thanks to its demonstrated bioactivity, CS has become an alternative in post-harvest food treatment to control the decomposition and extend shelf-life by the inhibitory effect against bacteria, fungi, and viruses. One of the feasible ways of applying CS has been as a film due to its filmogenic properties [18], allowing its proper tailoring in edible films and coatings. Hence, the following section addresses the relevant findings aimed at implementing CS in edible films and coatings with potential application into food packaging and preservation. To provide a better understanding in edible film and coating preparation, a background on the key characteristics required from materials for such applications is given.

## 3. Advances in Chitosan-Based Edible Films and Coatings

### 3.1. Key Characteristics of Edible Films and Coatings

Edible films and coatings are made of any biopolymeric material dispersed in an aqueous phase with additives (e.g., plasticizers and surfactants) [26]. Once the biomaterials are cast and dried, they may be able to form a sheet of less than 0.3 mm thick [27]. The main difference between an edible film and a coating deals with the fabrication mode [2,28]; for example, a typical edible film is fabricated on a mold (e.g., Teflon, glass, or ceramic) and it can be generated in monolayers (i.e., single biopolymer layer), multilayers (i.e., two or more biopolymers added separately), or as composite films (i.e., a mix of more than two biopolymers) using the solvent evaporation technique or casting [29]. On the other hand, edible coatings are directly applied on the product through three different scenarios: (i) by immersion [30], (ii) spread [31], or (iii) sprayed [32].

The materials used in edible films and coatings must have the ability to form rigid matrices [33,34], and thus be capable to protect the product from ultraviolet (UV)-light, and mechanical and pathogen damage; besides, they may display a controlled transport of gases (e.g., oxygen, carbon dioxide, and ethylene) and water vapor between the environment and the product, and vice versa. Together with all such features, the films and coatings must also maintain the original quality and nutritional characteristics of foods as long as possible and do not modify organoleptic properties [28,35,36,37]. Therefore, mechanical (e.g., elastic modulus, tensile strength, and elongation at break) and permeability properties are among the most important parameters to evaluate the functionality of edible films and coatings, since they indicate the resistance to product handling [38,39] and transfer of gases and vapors that can promote the growth of microorganisms and thus affect the maturation and senescence of food products [40]. Here, it is worth to highlight that the mechanical properties are not only highly influenced by the types of biopolymers, but also the addition of plasticizers (e.g., glycerol and sorbitol) [41] and surfactants (e.g., Tween 20 and Tween 80) [42]. By interacting with the biopolymeric materials, both agents (i.e., plasticizers and surfactants) cause a chemical modification of the three-dimensional matrix, specifically by increasing its hydrophobicity, leading to a reduction in interactions with water [43]. Permeability properties depend on the chemical structure, the nature of the gases, their solubility and diffusion across the polymer matrix, and handling conditions (i.e., temperature and pressure) [44,45]. Crucially, the morphological and structure properties of the edible films and coatings, which in turn depend on the preparation protocol used, greatly influence the transport of gases and vapors [46]. For instance, Table 2 elucidates some of the mechanical and permeability properties of polymers and biopolymers used in commercial food packaging and edible films, respectively.

### 3.2. Recent Advances in Chitosan-Based Edible Films and Coatings

As mentioned previously, CS is likely one of the most explored biopolymers in the manufacture of edible films and coatings. Table 3 summarizes the most updated development works aimed at using CS in the formulation of edible films and coatings over the last decade. Some authors have formulated and tailored CS-based edible films using different concepts of films, including monolayer, multilayer, and composites, varying the polysaccharide concentration and additives, as well as using different temperatures and drying technologies (e.g., irradiation, UV, or oven drying).

As a starting point, most of the research has been focused on evaluating different preparation conditions, incorporation of additives, polymer blending, embedding of bioactive compounds, natural extracts, among others, aiming to improve and extend the application of CS-based films and coatings. For instance, Homez-Jara et al. [7] evaluated the effect of the properties of CS-based films with different CS concentrations (from 0.5% up to 1.5% (*w*/*v*)) and drying temperatures (from 2 up to 40 °C). The moisture content (from 10.8% up to 31.3%) was mainly affected by drying temperature, which means that a higher temperature conducted to a lower moisture content. The effect on the mechanical properties (such as tensile strength and elongation at break) did not show a linear relationship with respect to the variation of CS concentration and temperature; however, the highest tensile strength (656 MPa) was obtained with the highest concentration and temperature (1.5% *w*/*v* and 40 °C, respectively) and the highest elongation at break (42.4%) occurred with a medium concentration and lowest drying temperature (1.0% *w*/*v* and 2 °C, respectively), while the water vapor permeability of the CS-based films decreased gradually (from 72.5 to 0.27 × 10^−11^ g m^−1^ s^−1^ Pa^−1^) with the increase of the CS concentration. Nevertheless, Zhang et al. [63] showed that the CS concentration (from 1% up to 2% *w*/*v*) influenced the increase in tensile strength (from 16 up to 28.5 MPa) and the decrease in elongation (from 10.5% down to 9.4%), while the increase in drying temperature (from 20 up to 80 °C) reduced the tensile strength (from 18.6 down to 12.2 MPa) and elongation (from 12.3% down to 9.7%). However, the authors noted that the addition of a plasticizer, such as glycerol (from 0% up to 1% w/w), decreased the water vapor permeability in CS-based films from 5 to 4.36 g·cm m^−2^·h^−1^·kPa^−1^ and water solubility about 25%. Interestingly, the application of a plasticizer does not always result in a beneficial factor on the mechanical and permeable properties of edible films and coatings, for example, the study reported by Farahnaky et al. [114] showed that wheat starch films became more brittle and less elastic with the addition of glycerol; furthermore, the permeability increased, possibly due to the structural modification generated between the starch and plasticizer, which caused a dense structure with greater movement, but less resistance.

The drying temperature and humidity are the main driving forces during the dehydration of materials and products [115]; however, the drying protocol in CS-based edible films has not been limited to such parameters. For example, Abraham et al. [49] noted that the intensity microwave radiation (from 60% up to 100%) had a greater influence than the application time (from 1 up to 5 min) in the drying CS-based edible films with vanillin (from 1% up to 4%), which was reflected in the increase of the swelling index from 321% up to 800%. Souza et al. [76], for instance, applied different electric field intensities (from 100 up to 200 V cm^−1^) and noted that higher electrical intensity resulted in a greater crystallinity of the films, and consequently improved the tensile strength and elongation at break. This is, perhaps, due to the fact that the energy emitted modifies the molecule charges, allowing more physical, chemical, and hydrogen bonds between molecules, and thus a strong three-dimensional matrix is generated.

Regardless of the drying method (by oven, microwave radiation, or electric fields) or the film-forming materials (e.g., beeswax) [116], several studies have shown that the faster drying of edible films generates usually more homogeneous structures and with greater crystallinity, while it has been documented that crystallinity promotes and improves mobility and mechanical properties [99,105].

To extend the application and functionality of the CS-based films and coatings, the incorporation of biologically active compounds extracted from naturals extracts into CS film formulations has been reported. The use of plants and their extracts has been carried out empirically since ancient times, particularly for the treatment of infectious diseases; however, it was later discovered that the alleviation of symptoms was linked to the antimicrobial and antioxidant activity of such bioactive molecules [117]. Active compounds can be obtained directly from plenty of natural sources, such as propolis, mint, thyme, rosemary, cinnamon, boldo-do-chile, guarana, murta, grape, and pomegranate, to mention just a few of them. Such biologically active compounds can be extracted by means of organic solvents extractions (e.g., water, ethanol, and methanol) [118], membrane processes [119], among other emerging extraction technologies (e.g., high-voltage electrical discharges, microwave-assisted extraction, ultrasound-assisted extraction, and pulsed ohmic heating) [120]. Once the active compounds have been successfully extracted, their importance relies on their potential antioxidant activity together with inhibitory effect against bacteria and fungi [121], especially phenolic compounds (e.g., catechin, rutin, kaempferol, and chlorogenic, caffeic, and ferulic acids) [122]. Importantly, the characteristics and properties of the CS-based edible films and coatings may change with the addition of the natural extracts. For example, Siripatrawan and Vitchayakitti [55] observed that propolis extract reduced the permeability to water vapor (about 28%) and oxygen (about 62%) but improved the tensile strength (about 266%) and elongation at break (about 140%) of CS-based films. Interestingly, the enhanced mechanical properties can be substantially related to a possible cross-linking effect [123], e.g., it has been reported that a strong interaction between the CS and phenolic components, such as quercetin, naringenin, and baicalin [124], can potentially produce a crosslinking reaction, resulting in a compact structure of the resulting edible films [77]. In addition to this, the phenolic compounds of the extract were possibly responsible for antimicrobial and antioxidant properties [55]. Concurrently, Rubilar et al. [43] also observed that CS-based films with grape seed extract (from 160 up to 684 ppm) and carvacrol (from 9.6 up to 90 ppm) decreased the physical properties (water solubility by 9% and moisture content by 67%), water vapor permeability (about 48%), and mechanical properties (tensile strength by 67% and elongation at break by 145%) in direct relation to the carvacrol concentration; unfortunately, this negative reduction was possibly attributed to the mobility decrease of the polymer matrix by the carvacrol hydrophobicity [57]. On the contrary, the incorporation of natural extracts has been done in different studies, confirming the improvement of CS-based films in terms of tensile strength and antimicrobial activity against *Staphylococcus aureus* and *Bacillus cereus*, as reported by Kanatt et al. [65], who added peppermint and pomegranate extracts into CS.

Essential oils are defined as complex mixtures of more than 300 volatile chemical compounds with low molecular weight (<1000 Da) [125], and they have also been recognized due to their healing power towards diseases transmitted by pathogenic microorganisms [126]. Positively, their popularity has recently increased in the food industries and research areas since they are recognized as a green alternative of additives that improve the quality of food and perishable products [127]. Some studies have demonstrated that essential oils contained in different parts of the plant, such as flowers, leaves, fruits, roots, stems, and seeds [128], exhibit antimicrobial, analgesic, sedative, anti-inflammatory, spasmolytic, anesthetic, and antioxidant effects [129]. Nowadays, the tuning of characteristics and properties of edible films and coatings by the addition of essential oils in formulations is continuously evaluated.

Avila-Sosa et al. [67] proved a better antifungal effect against *Aspergillus niger* and *Penicillium digitatum* in CS-based films by enriching with essential oils from oregano or cinnamon. While, Hafsa et al. [53] found that the eucalyptus essential oil showed antiradical activity, especially against 2,2-diphenyl-1-picrylhydrazyl (DPPH), nitric oxide, and hydrogen peroxide in CS-based films. More recently, Peng and Li [61] noted that lemon, thyme, and cinnamon essential oils inhibited *Escherichia coli* by 474%, 1969%, and 1895% respectively, and *Staphylococcus aureus* by 575%, 3619%, and 2427%, respectively. Additionally, essential oils slightly decreased the water vapor permeability by 5.3%, 2.4%, and 5.0% respectively, while the water solubility was decreased by 51%, 28%, and 34%, respectively. The elongation at break was also negatively affected by 54%, 31%, and 42% respectively, but the essential oils increased the opacity in CS-based films by 601%, 272%, and 281%, respectively. When essential oils were blended in mixture, some studied properties were significantly affected; for example, the tensile strength, opacity, and water solubility decreased by about 25%, 784%, and 26% respectively, but the antimicrobial activity against *Escherichia coli* and *Staphylococcus aureus* increased by 1511% and 2089%, respectively. The inhibition is thanks to the phenolic content of essential oils since they are responsible for neutralizing reactive species and interacting with the membrane components of microorganisms by the contribution of electrons [130,131].

As a current trend in the field, the design for tailoring single, composite, or multilayer films is a latent way of film fabrication. Pereda et al. [85] generated two types of composite and bilayer films based on CS and bovine gelatin. Basically, the authors dispersed both biopolymer phases to generate composite films, while a general multilayer methodology was also used to generate bilayer films. Theoretically, the dispersions were made separately by casting technique, one dispersion is dried before pouring the next one [58]. According to Pereda’s findings, both concepts of films presented smooth and homogeneous surface; however, the bi-layered films displayed an interface between layers, which indicated a lack of compatibility, compactness, and adhesion among biopolymers [85]. Both films showed similar tensile at break (6.27 and 8.55 MPa, respectively) but the composite films had especially a higher percentage of elongation at break (85.4% against 41.0%); in addition to this, the inhibitory power of both films against *Escherichia coli* was not affected. Unlike Pereda’s work [85], Zhang et al. [80] reported good compatibility and improvement in properties of copy paper covered by a bilayer films based on CS and beeswax, specifically in the decrement of the water vapor permeability (up to 98%). This was attributed to the viscoelasticity of beeswax since it allowed the adhesion of CS and copy paper while the malleability of the biopolymeric matrix allows the transport of vapor [132]. In this study, the authors confirmed the excellent capacity of CS as an emulsifier to prepare stable lipid latex with beeswax thanks to the electrostatic interaction between positively charged CS and negatively charged lipids (carboxyl groups) of beeswax [133].

### 3.3. Applications of Chitosan-Based Edible Films and Coatings on Food Products Models

In principle, the main objective of using edible films and coatings in food products pursues their preservation, serving as an alternative to conventional packaging [33]. Of course, the most challenging task of edible films and coatings deals with the preservation of the natural characteristics of foods and delays spoilage due to physical damage, and chemical and biochemical reactions [134]. Such targets have been successfully achieved by edible films and coatings based on CS, for instance, Table 4 summarizes outstanding studies in which several food products (e.g., fruits, vegetables, animal products, and dairy) have been preserved with CS-based edible films and coatings, highlighting the meaningful impact of using such materials in the productsʹ quality.

By keeping in mind that food spoilage is influenced by oxygen, water content, temperature, relative humidity, and pH [176], the physicochemical and mechanical properties of the films should efficiently control such storage conditions, and as a consequence, maintain the physical and sensory characteristics together with the protection against microbiological pathogens and increase foods’ shelf-life [177]. This becomes more important in perishable natural foods (e.g., fruits and vegetables), which continue to metabolize and breathe after harvest, which means they consume oxygen to produce carbon dioxide, ethylene, and water (in different proportions), which causes the product ripening and later its decomposition [178], whereas the decomposition in perishable foods of animal origin (e.g., meat and seafood) begins immediately after death because they are highly susceptible to microbial attack by *Staphylococcus aureus*, *Listeria monocytogenes*, and *Salmonella* [179]. For this reason, the application of edible films and coatings is mostly directed towards highly perishable products to preserve their quality (e.g., taste, texture, and appearance) for a longer time.

Apriyanti et al. [137] applied a functional CS film enriched with green tea extract for the protection of strawberries. Since such natural extract contains considerably high amounts of phenolic compounds, they noted an increase in the phenolic content according to the gallic acid equivalents (GAE) from 3.5 up to ~14 mg GAE g^−1^, as well as increased antioxidant capacity from 5% up to ~70%, which in fact was dependent on the concentration of CS and tea extract. Similarly, using strawberries as a model, Perdones et al. [148] proved that the properties of the strawberries did not change significantly by packing with CS coatings containing lemon essential oil; more importantly, lemon essential oil successfully extended shelf-life of the strawberries by mitigating the fungal attack. In another investigation, Vu et al. [158] covered strawberries with a film-forming solution of CS and different essential oils extracted from limonene, oregano, thyme, peppermint, or lemongrass. Practically, the authors noted an extension of fruits’ shelf-life for over double the time (14 days); in addition, the greatest inhibitory effect on the native flora (43.3 mm in inhibition diameter) and molds (57.5 mm in inhibition diameter) were achieved when using red thyme essential oil, and the least effect (0.0 and 15.5 mm in inhibition diameter, respectively) was generated by limonene essential oil. Although the authors did not precisely state the reason for the efficacy of red thyme essential oil, they attribute it to its antifungal power that has been previously studied on native flora from strawberries (against *Botrytis cinerea* and *Rhizopus stolonifer*) [180].

More recently, Yusof et al. [165] coated strawberries with a complex edible coating based on a CS-corn starch blend enriched with *Curcuma longa* L. essential oil. A good interaction between the two biopolymers was improved by the essential oil addition; in general, the coatings inhibited the native flora of the fruit and concurrently extended its shelf-life for 5 days without refrigeration. Similarly, Azevedo et al. [166] used CS-based edible coatings formulated with cassava starch and *Lippia gracilis* essential oil to protect strawberries, noting that the essential oil increased the antimicrobial and antifungal effect against yeasts, molds (up to 99.8%), and psychotropic bacteria (up to 98.7%), maintaining a bacteriostatic effect on total and thermo-tolerant coliforms according to the most probable number (<3 MPN g^−1^).

By preparing a composite film, Velickova et al. [82] used three-layer coatings of beeswax/CS/beeswax to preserve strawberries. The addition of the wax improved the tensile strength and elastic modulus of the coating, while maturity index of the fruit decreased due to control of respiration rate and fungal infection, with the latter attributed to beeswax. It is important to mention that thanks to its rich hydrophobic protective properties, the beeswax is indeed used in cosmetics and body products [181]. The bilayer effect was also evaluated by Nowzari et al. [162], who formulated a film-forming solution with CS and fish gelatin to protect rainbow trout. This study evaluated the food protection in two different scenarios, (i) by dipping one polymer followed by the other, and (ii) by wrapping with film in pre-made bilayer. The authors were monitoring the free fatty acids content over a period of 16 days; in theory, these are the result of enzymatic hydrolysis of esterified lipids (e.g., phospholipids and triglycerides), promoting oxidation reactions with proteins [182,183]. The results showed a greater slowdown in the free fatty acids formation using bilayer films and the same inhibitory effect (of about 40%) of native pathogens with both coatings [162].

In some other perishable products, such as cheeses and meats, the nutritional content and the water content are the most sensitive parameters during their storage since they allow the proliferation of microorganisms and the degradation of the product [184]. In the light of cheese preservation, Brown et al. [136] protected fresh cheese using a coating based on CS, hydrogen peroxide, lauric arginate, and calcium sulfate. Until now, it has been well-proven the efficiency of CS to extend the shelf-life of food products and control the action of pathogens. The embedding of different antimicrobials, such as essential oils, enzymes, and bioactive compounds, has also provided an enhanced activity towards the growth of *Listeria monocytogenes*, however, simple additives and chemicals, such as lactic acid, hydrogen peroxide, lauric arginate, and sodium caprylate, have also been recognized for their effectiveness in inhibiting *Listeria monocytogenes* in broth and whole milk. Therefore, Brown and co-workers analyzed the further antimicrobial effect of hydrogen peroxide, lauric arginate, and calcium sulfate into CS when utilized before or after surface contamination at refrigerated storage. After evaluation, the authors concluded that such formulated films successfully controlled the growth of *Listeria monocytogenes* and extended the shelf-life of the cheese up to 35 days with refrigeration. Fajardo et al. [156] used CS-based edible films containing natamycin, which was subsequently implemented to protect Saloio cheese against *Aspergillus niger*. By releasing the fungicide, a suitable inhibition of the strain was reached, controlling its growth, and the shelf-life increased from 7 days (at 25 °C) up to 37 days (at 4 °C). In previous studies, the success has been mainly attributed to the additives that synergistically improved the biological activity of CS acting against pathogenic organisms. CS also offers the possibility to be blended with protein materials, as shown by Di Piero et al. [173]. Here, the authors combined CS and whey protein to fabricate films for the conservation of ricotta cheese. These proposed films delayed undesirable acidity, improved texture, maintained sensory characteristics, and inhibited mesophilic and psychotropic bacteria at modified atmosphere of CO_2_/Nitrogen (N_2_).

Ruiz-Cruz et al. [135] have very recently increased the chicken fillets shelf-life over 16 days due to the inhibition of mesophilic, psychophilic, and coliform bacteria by means of CS-based edible coatings enriched with tomato extract, which acted as an antimicrobial and antioxidant compound. On the other hand, Karimnezhad et al. [138] achieved a greater reduction of aerobic, psychrophilic, and coliform bacteria for 12 days, maintaining the odor, color, taste, texture, and acceptability of the chicken meat. Such benefits were related to the excellent action and protective effect of CS films loaded with *Trachyspermum ammi* extract.

## 4. Concluding Remarks

Bio-packaging made from biodegradable materials is a current trend in the field of preservation of food products, however, it is still an important challenge for food technicians since the materials must meet specific requirements to protect food products. CS-based edible films and coatings have successfully demonstrated their ability to fabricate different concepts of monolayer or composite films.

The ability of CS to form edible films is mainly due to its polycationic nature that allows it to work in conjunction with other biopolymers and additives. This synergy causes strong physical interactions between all the components, which reflects the improvement of the physical, mechanical, and permeability properties. In addition, the bioactive potential of CS-edible films is originated by its intrinsic properties such as antimicrobial, antifungal, and antioxidant capacities. These features make CS potentially interesting compared with other biomaterials.

The evidence on CS-edible films and coatings shows that they meet the requirements to be part of the so-called "green" bio-packaging and be an alternative to synthetic packaging. However, this type of technology is highly targeted towards characteristics that must be adequate and specific for the type of food to be protected, making the area of edible films or coatings have a great potential for study. Finally, since several bioactive compounds extracted from natural sources are providing various benefits to human health, it is likely that CS will continue to be explored as a support for the incorporation of new functional edible films.

## 5. Future Perspectives

Beyond the ecological aspect that CS represents within the food and packaging industries when used for "green" bio-packaging, it must be specified that it is a highly important polysaccharide due to its availability in nature to acquire it. For instance, it is believed that the global chitin and chitosan market could reach $4.2 billion by 2021 from $2.0 billion in 2016 at a compound annual growth rate (CAGR) of 15.4% [185], while most of the new environmentally friendly materials are still not consolidated. At this point, CS has been widely studied and proven in plenty of applications, and currently has meaningful interest in medicine, cosmetics, the food industry, and biotechnology [186]. Moreover, it has shown its ability to form sustainable films, and more interestingly, it is able to interact with other polymeric materials, resulting in new blends with enhanced properties. CS has also demonstrated its ability to incorporate a number of bioactive components in its matrix. We believe that CS will still be explored for its ability in carrying bioactive compounds toward functional bio-packing manufacture. Nevertheless, these industries are in constant biotechnological renovation to market better quality food products with better benefits to the consumer, where the implementation of CS is fostered for intelligent packaging.

Today, intelligent packaging is a complex system generated mostly from petroleum-based plastics according to available development works. However, trends clearly show that bio-packaging will also improve to become part of these systems to monitor, generate, and display relevant information about the product. Therefore, the nature of CS and its compatibility with various materials make it a candidate to evolve into these new packaging technologies.

## Figures and Tables

**Table 1:** Pathogens inhibited by the action of chitosan.

Types of Pathogens	Examples	References
Bacteria		
Gram-positive	*Bacillus* spp. *Listeria monocytogenes* *Staphylococcus aureus*	[23]
Gram-negative	*Aeromonas hydrophila* *Escherichia coli* *Pseudomonas aeruginosa* *Salmonella typhymurium* *Shigella dysenteriae* *Vibrio cholera* *Vibrio parahaemolyticus*	
Fungi		
Phytopathogens	*Alternaria alternata* *Aspergillus niger* *Botrytis cinerea* *Candida albicans* *Colletotrichum spp.* *Fusarium oxysporum* *Lasiodiplodia theobromae* *Monilinia fructicola* *Monilinia laxa* *Penicillium spp.* *Physalospora piricola* *Rhizoctonia solani* *Rhizopus stolonifer* *Sclerotinia sclerotiorum* *Verticillium albo-atrum*	[18]
Virus		
Phytopathogens	Alfalfa mosaic virus Bean goldish mosaic virus Peanut stunt virus Tobacco mosaic virus Potato virus X and Y Figwort mosaic virus Cucumber mosaic virus Bean common mosaic virus Potato spindle tuber viroid	[24]

**Table 2:** General properties of polymers and biopolymers used in food packaging.

Packaging Materials	Mechanical Properties	Barrier Properties	References
Polymer materials *			
Low-density polyethylene	Tough and flexible	High moisture barrier and very low gas barrier	[47]
Linear low-density polyethylene	Tough and extensible	High moisture barrier and very low gas barrier	
High-density polyethylene	Tough, stiff, strong, and easy to form	Extremely high moisture barrier and very low gas barrier	
Polypropylene	Moderately stiff and strong	High moisture barrier and low gas barrier	
Polyesters	High impact-resistance, low scratch-resistance, tough and strong	High barrier to gases and moisture	
Polyethylene terephthalate	Stiff and strong	Good barrier to gases and moisture	
Polyethylene naphthalate	Stiff	Good barrier to gases and moisture	
Polyvinyl chloride	Strong and stiff ductile	High moisture barrier and moderate oxygen barrier	
Polyvinylidene dichloride	Strong and stiff ductile	Excellent barrier to oxygen and moisture	
Polystyrene	Hard and brittle	Low barrier to moisture and air	
Ethylene vinyl alcohol	Stiff and strong	Excellent moisture barrier and high air barrier	
Polyamide	Stiff and strong	High barrier to air and moisture	
Biopolymer films **			
Cellophane	Good	Moderate moisture barrier and good oxygen barrier	[48]
Cellulose acetate	Good	Moderate moisture barrier and poor oxygen barrier	
Polylactic acid	Good	Moderate moisture barrier and poor oxygen barrier	
Methyl cellulose	Moderate	Moderate barrier to moisture and oxygen	
Hydroxypropyl methylcellulose	Moderate	Moderate barrier to moisture and oxygen	
Gelatin	Not applicable	Poor moisture barrier and good oxygen barrier	
Zein	Moderate	Moderate barrier to moisture and oxygen	
Gluten	Moderate	Moderate moisture barrier and good oxygen barrier	
Soy protein isolate	Moderate	Poor moisture barrier and good oxygen barrier	
Casein	Not applicable	Poor moisture barrier and good oxygen barrier	
Whey protein isolate	Moderate	Poor moisture barrier and good oxygen barrier	

* Conditions are based on commercial packaging; ** Conditions depend on the plasticizer and the thickness of the packaging material.

**Table 3:** Chitosan (CS)-based edible films and coatings.

Biopolymeric Matrices	CS Properties	Additives	Outstanding Results	References
Monolayer				
CS	15% DA	Vanillin Acetic acid	Microwave irradiation was a rapid, reliable, and economic method cross-linking	[49]
CS	50–190 kDa MW; 15–25% DA	Acetic acid Tween 80 Glycerol	Improvement of water vapor permeability, solubility, tensile strength, and optical properties	[7]
CS	Medium MW; 15% DA	Pistacia extracts Acetic acid Glycerol	Antioxidant activity improvement Inhibition of *Escherichia coli*, *Staphylococcus aureus,* and *Salmonella*	[50]
Quaternized carboxymethyl CS	16% DA	Polyvinyl alcohol Copper sulfate	Mechanical and thermal properties improvement Inhibition of *Staphylococcus aureus* and *Escherichia coli*	[51]
CS	190–310 kDa MW; 15–25% DA	Silybum marianum extract Sodium montmorillonite Acetic acid Glycerol	Improvement of water vapor permeability, water resistance, antioxidant activity, and mechanical properties	[52]
Shrimp-CS	<25% DA	Eucalyptus essential oil Acetic acid Glycerol	Improvement of antioxidant and antimicrobial activities	[53]
CS	High MW; 22% DA	Thyme essential oil Basil essential oil Oleic acid Acetic acid	Improvement of emulsion stability and water vapor permeability	[54]
CS	5% DA	Propolis extract Glycerol Acetic acid	Improvement of mechanical properties, permeability, and antimicrobial and antioxidant activities	[55]
CS	High MW; 5% DA	Arabinoxylans Arabino-xylo-oligosaccharides Lactic acid Tween 80 Glycerol	Improvement of mechanical and prebiotic properties	[56]
CS	400 kDa MW 180 kDa MW 41 kDa MW	Carvacrol Acetic acid Glycerol	Inhibition of *Pseudomona fragi*, *Shewanella putrefaciens,* and *Aeromonas hydrophila* Carvacrol release was faster with CH of high MW	[57]
CS	165 kDa MW; <15% DA	Carvacrol Acetic acid	Gas permeability improvement Carvacrol release was faster with relative humidity	[58]
CS	800 kDa MW; 2–5% DA	Gallic acid Glycerol Acetic acid	Antimicrobial activity improvement Tensile strength increment Reduction of water vapor and oxygen permeability	[59]
CS	Low MW; 15–25% DA	Lysozyme Lactic acid Glycerol	Inhibition of *Listeria monocytogenes* and *Pseudomonas fluorescens*	[60]
Crab-CS	10% DA	Lemon essential oil Thyme essential oil Cinnamon essential oil Acetic acid Tween 20 Glycerol	Decrement of water vapor permeability and elongation Improvement of barrier properties, emulsion stability, particle size, and viscosity	[61]
CS	5–20% DA	Tea polyphenols Acetic acid Glycerol	Improvement of water vapor permeability and antioxidant activity	[62]
CS	-	Acetic acid Glycerol	Improvement of mechanical and permeability properties	[63]
CS	High MW; <25% DA	Carvacrol Grape seed extract Acetic acid Tween 80 Glycerol	Decrement of water vapor and carbon dioxide permeabilities, tensile strength, and elongation at break Increment of water content and oxygen permeability	[43]
CS	High MW; 15–25% DA	Thyme essential oils Lactic acid Tween 80 Glycerol	Improvement of antibacterial and antioxidant activities	[64]
CS	186 kDa MW; 10% DA	Polyvinyl alcohol Mint extract Pomegranate extract Acetic acid Glycerol	Tensile strength improvement Gram-positive bacteria inhibition	[65]
CS	161 kDa MW; 10% DA	Olive oil Acetic acid Glycerol	Increment of tensile strength and elongation at break Decrement of moisture sorption and water vapor permeability	[66]
CS	450 kDa MW	Oregano essential oil Cinnamon essential oil Lemongrass essential oil Acetic acid Tween 20 Glycerol	Inhibition of *Aspergillus niger* and *Penicillium digitatum*	[67]
CS	10% DA	Corn oil Sorbitol Lactic acid Tween 80 Glycerol	Improvement of permeability, and mechanical and optical properties	[68]
CS	<10% DA	Lactic acid Glycerol	Improvement of carbon dioxide and water vapor permeabilities, and mechanical properties	[69]
CS	5% DA	ɑ-Tocopherol Lactic acid Tween 80	Improvement of antioxidant activity and water vapor permeability Reduction of tensile strength and elongation at break	[70]
CS	450 kDa MW; 15–25% DA	*Zataria multiflora* essential oil Grape seed extract Acetic acid Glycerol	Improvement of water vapor permeability, phenolic content, and antioxidant activity	[71]
CS	High MW	Thyme essential oil Basil essential oil Acetic acid	Improvement of water vapor permeability and optical characteristics	[72,73]
CS	450 kDa MW	Oregano essential oil Acetic acid Tween 20	Inhibition of *Aspergillus niger* and *Penicillium* spp.	[74]
CS	High MW; 17% DA	Bergamot essential oil Acetic acid	*Penicillium italicum* inhibition Water vapor permeability improvement	[75]
CS	10% DA	Lactic acid Tween 80	Improvement of crystallinity, structure, tensile strength, and elongation at break	[76]
Crab-CS	190–310 kDa MW; 15–25% DA	Cinnamon essential oil Acetic acid Tween 80 Glycerol	Improvement of antibacterial, physical, and mechanical properties	[77]
Shrimp-CS Crab-CS	100 kDa MW; 24% DA 480 kDa MW; 35% DA	Sorbitol Acetic acid	Increment of thermal stability, fungal activity, and mechanical properties	[78]
**Multilayers (chitosan + other biopolymers)**				
CS/Pullulan (composite film)/Polyethylene	-	Glycerol Lactic acid	Oxygen permeability improvement Inhibition of *Staphylococcus aureus* and *Aspergillus flavus*	[79]
CS/Beeswax (composite film)/Copy-paper	Medium MW; 15–25% DA	Acetic acid Glycerol	Water vapor permeability improvement	[80]
CS/Whey protein isolate	165 kDa MW; <15% DA	Acetic acid Glycerol	Improvement of water vapor permeability and mechanical properties	[81]
CS/Beeswax Beeswax/CS/Beeswax	<15% DA	Sodium tripolyphosphate Acetic acid Glycerol Tween 80	Increment of water vapor permeability and stiffness	[82]
CS/Wheat starch	High MW	Basil essential oil Thyme essential oil Citric acid ɑ-Tocopherol Glycerol	Structural change induction Antioxidant effect increment Improvement of water vapor permeability and mechanical properties	[83]
CS/Polyethylene film	165 kDa MW; 15% DA	Acetic acid Glycerol	Decrement of water vapor and gas permeabilities	[84]
CS/Bovine gelatin	10% DA	Acetic acid Glycerol	Reduction of solubility and water vapor permeability Mechanical properties improvement Inhibition of against *Escherichia coli* and *Listeria monocytogenes*	[85]
**Composite film (biopolymers mixture)**				
Modified CS/Nanocrystalline cellulose	15% DA	Acetic acid	Mechanical properties improvement *Pseudomonas Aeruginosa* inhibition	[86]
CS/Cellulose nanofiber CS/Lignocellulose nanofiber	190–310 kDa MW; 15–25% DA	*Carum copticum* essential oil Acetic acid Tween 80 Glycerol	Improvement of solubility, water vapor permeability, and mechanical properties	[87]
CS/Pea starch	High MW	Thyme extract Tannic acid Acetic acid Glycerol	Improvement of antioxidant activity and mechanical properties	[88]
CS/Corn starch	30% DA	Lauric acid Acetic acid Glycerol	Tensile strength increment Decrement of elongation and water vapor permeability Gram-positive bacteria inhibition	[89]
CS/Fish gelatin	165 kDa MW; 15% DA	Quercetin Acetic acid Glycerol	Structure modification Control of quercetin release	[90]
CS/Pigskin gelatin	Medium MW; 15–25% DA	Rosemary extract Cinnamon extract Boldo-do-chile extract Guarana extract Glycerol	Water vapor permeability decrement Improvement of mechanical properties and antioxidant and antimicrobial activities	[91]
Crab-CS/Corn starch	Low MW; 15% DA Medium MW; 15% DA High MW; 15% DA	Acetic acid Glycerol	Improvement of structure, water vapor permeability, elasticity, solubility, and resistance by CH of high MW	[92]
CS/Fish gelatin	3-449 kDa MW; 20–25% DA	Transglutaminase Acetic acid Glycerol	Structural change induction Mechanical properties increment	[93]
CS/Tara gum CS nanoparticles/Tara gum	100 kDa MW; 10% DA	Acetic acid Glycerol	Improvement of moisture, water vapor permeability, and mechanical properties	[94]
CS/Zein	50–190 kDa MW; 10% DA	Gallic acid Ferulic acid Succinic acid Adipic acid Acetic acid Glycerol	Improvement of mechanical properties, water vapor permeability, and antioxidant and antimicrobial activities	[95]
CS/Potato starch CS/Cassava starch	149 kDa MW; 10% DA	Acetic acid Glycerol (or glucose) Tween 20 (or 80)	Increment of tensile strength, elasticity modulus, solubility, and water vapor permeability	[96]
CS/Tapioca starch	900 kDa MW; 15% DA	Graphene platelets Acetic acid Glycerol	Improvement of water vapor permeability and mechanical and thermal properties	[97]
CS/Zein	<25% DA	Acetic acid Glycerol	Improvement of water vapor permeability and mechanical properties	[98]
CS/Fish gelatin	Medium MW; 15–25% DA	Acetic acid Glycerol	Improvement of tensile strength, elastic modus, and water vapor permeability	[99]
CS/Methylcellulose	15–25% DA	Resveratrol Acetic acid	Structural change induction Antioxidant activity improvement	[100]
CS/Soy protein isolate	-	Palm stearin Glycerol	Thermoplasticity improvement	[101]
CS/Corn starch	190–310 kDa MW; 25% DA	Murta leaves extract Acetic acid Glycerol	Polymer mobility induction Mechanical properties improvement	[102]
CS/Silk fibroin	Low MW; 20% DA	Acetic acid	Roughness decrement	[103]
Crab-CS/Quinoa flour	15–25% DA	Sunflower oil Citric acid Tween 80	Improvement of water vapor permeability and mechanical properties	[104]
CS/Pullulan Carboxymethyl CS/Pullulan	300 kDa MW; 15% DA	Acetic acid Glycerol	Induction of strong interactions, stability, and flexibility	[105]
CS/Wheat starch	High MW; <23% DA	Acetic acid Glycerol	Improvement of surface charge, the apparent viscosity, bright, thickness, resistance, elongation, water vapor and oxygen permeabilities, and antimicrobial activity	[83]
CS/Sodium alginate	Low MW; 15–75% DA	Acetic acid Glycerol	Efficient evaluation of microstructure by microscopic techniques	[106]
Crab-CS/Corn starch	400 kDa MW; 15–25% DA	Thyme essential oil Acetic acidTween 80 Glycerol	Improvement of antimicrobial and antioxidant activities	[107]
CS nanoparticles/Carboxymethylcellulose	71 kDa MW; 6% DA	Methacrylic acid	Water vapor permeability decrement Improvement of mechanical properties and thermostability	[108]
CS/Quinoa protein	408 kDa MW; 23% DA	Lactic acid	Improvement water vapor permeability, and mechanical and thermal properties	[109]
CS/Whey protein concentrate CS/Glycomacropeptide CS/Lactoferrin	10% DA	Lactic acid Tween 80 Glycerol	Decrement of gas permeability and tensile strength Elongation at breakage increment	[110]
CS/Cellulose nanofibers	71 kDa MW; 6% DA	Acetic acid Glycerol	Improvement of mechanical and water vapor permeability properties	[111]
CS/Pectin	21% DA	Polyvinyl alcohol Acetic acid	Pathogenic bacteria inhibition	[112]
CS/Sodium cellulose sulfate	118.7 kDa MW; 15% DA 135.3 kDa MW; 15% DA 563.3 kDa MW; 15% DA 723.2 KDa MW; 15% DA	-	Biodegradation by pepsin, amylase, and trypsin	[113]

CS: chitosan; MW: molecular weight; DA: degree of acetylation.

**Table 4:** Application of chitosan-based edible films and coatings on food products.

Biopolymeric Matrices	Additives	Coating Techniques	Models	Results	References
CS	Tomato plant extract Acetic acid Glycerol	Dipped	Chicken	Quality parameters conservation Microbial inhibition Shelf-life prolongation	[135]
CS	Lactic acid Hydrogen peroxide Lauric arginate Sodium caprylate	Dipped	Fresh cheese	*Listeria monocytogenes* inhibition	[136]
CS	Green tea extract Acetic Glycerol	Dipped	Strawberries	Total phenolic content increase Increment of antioxidant and antimicrobial activities	[137]
CS	Trachyspermum ammi extract Acetic acid Tween 80 Glycerol	Wrapped	Chicken meat	Microbial inhibition Shelf-life prolongation	[138]
CS	Acetic acid Glycerol	Dipped	Apples	Conservation of weight, water content, and vitamin C	[139]
CS	Acetic acid Glycerol	Sprayed Dipped	Baby carrots	Microbial inhibition	[140]
CS	*Thymus piperella* essential oil *Thymus moroderi* essential oil Lactic acid Tween 80 Glycerol	Wrapped	Cooked cured ham	Bacterial inhibition Lipid oxidation protection Shelf-life prolongation	[141]
CS	n.r.	Dipped	Loquats	Conservation of polyphenols, flavonoids, ascorbic acid, and carotenoids Senescence delay	[142]
CS	Ascorbic acid Citric acid	Dipped	Plums	Reduction of weight loss, respiration rate, and stress Firmness conservation	[143]
CS	Garlic oil Acetic acid Glycerol	Dipped	Raw shrimp meat	Nitrogen content reduction Shelf-life prolongation	[144]
CS	Acetic acid Glycerol	Wrapped	Fresh sea bass	Bacterial inhibition Shelf-life prolongation	[145]
CS	Acetic acid Tween 80	Dipped	Guavas	Ripening process delay Oxidative stress decrease	[146]
CS	Acetic acid	Dipped	Indian oil sardine	Sensory properties conservation Oxidation compounds reduction Shelf-life prolongation	[147]
CS	Lemon essential oil Acetic acid	Dipped	Strawberries	Fungal inhibition Shelf-life prolongation	[148]
CS	Palm stearin Acetic acid Tween 80	Dipped	Star fruits	Weight loss reduction Conservation of firmness, appearance, and quality	[149]
CS	Zataria multiflora essential oil Grape seed extract Acetic acid Glycerol	Wrapped	Ready-to-eat mortadella sausages	Shelf-life prolongation	[150]
CS	Acetic acid Glycerol	Dipped	Broccoli	Bacterial inhibition Shelf-life prolongation	[151]
CS	Acetic acid	Dipped	Apples	Fungal inhibition	[152]
CS	Sunflower oil Acetic acid (or lactic acid)	Wrapped	Pork meat hamburgers	Shelf-life prolongation	[153]
CS	Sodium lactate Sodium diacetate Potassium sorbate Acetic acid	Wrapped	Cold-smoked salmon	*Listeria monocytogenes* inhibition	[154]
CS	Fish oil Vitamin E500 Acetic acid Tween 80 Glycerol	Dipped	Lingcod	Acid-reactive substances reduction Bacterial inhibition Conservation of lipids, omega-3 fatty acids, and fatty acidsShelf-life prolongation	[155]
CS	Natamycin Acid lactic Tween 80 Glycerol	Wrapped	Saloio cheese	*Aspergillus niger* inhibitionShelf-life prolongation	[156]
CS silver nanoparticles	Glucose	Spread (four-layer)	Tomatoes Apples	Shelf-life prolongation	[157]
Modified CS	Limonene essential oil Oregano essential oil Red thyme essential oil Peppermint essential oil Lemongrass essential oil Acetic acid Tween 80	Sprayed	Strawberries	Native pathogens inhibition Shelf-life prolongation	[158]
N, O-carboxymethyl CS	Sodium chlorite Acetic acid	Dipped	Pears	*Escherichia coli* inhibition	[159]
CS-Gelatin	Mint essential oil Thyme essential oil Acetic acid	Wrapped	Black radish	*Listeria monocytogenes* inhibition	[160]
CS/Bovine gelatin	Glycerol	Dipped	Beef	Reduction of weight loss, lipid oxidation, and discoloration Shelf-life prolongation	[161]
CS/Fish gelatin	Acetic acid Glycerol	Wrapped (bilayer) Dipped (bilayer)	Rainbow trout	Bacterial inhibition Antioxidant effects increase Lipid oxidation delay	[162]
CS/Bovine gelatin	Clove essential oil Soy lecithin Acid lactic Sorbitol Glycerol	Wrapped	Cod	Microorganisms inhibition	[163]
CS/Quinoa protein	High oleic sunflower oil Lactic acid	Dipped	Blueberries	Conservation of firmness and color Mold inhibition Shelf-life prolongation	[164]
CS/Corn starch	Curcuma essential oil Tween 80 Acetic acid Glycerol	Dipped	Strawberries	Microbial inhibition Ripening and spoilage delay	[165]
CS/Cassava starch	Essential oils (from *Lippia gracilis* Schauer) Acetic acid Tween-80 Glycerol	Dipped	Strawberries	Gram-positive bacteria inhibition Shelf-life prolongation	[166]
CS/Mung bean starch CS/Water chestnut starch	Perilla oil Acetic acid Glycerol	Spread	Mongolian cheese	Inhibition of bacteria and fungi Weight loss delay Shelf-life prolongation	[167]
CS/Kappa carrageenan	Sunflower oil Acetic acid Glycerol	Dipped	Longan fruits	Shelf-life prolongation	[168]
CS/Alginate	Acetic acid Calcium chloride	Dipped (bilayer)	Melons	Inhibition of bacteria, yeasts, and molds Firmness conservation	[169]
CS/Carboxymethyl cellulose (bilayer)	Acetic acid	Spread	Mandarins, oranges and grapefruits	Glossiness and appearance improvement	[170]
Beeswax/CS/Beeswax (three layer)	Sodium tripolyphosphate Acetic acid Glycerol Tween 80	Dipped	Strawberries	Native fungi inhibition Shelf-life prolongation	[82]
CS/O-carboxymethyl CS	Acetic acid	Dipped	Whiteleg shrimp	Microbial spoilage delay Shelf-life prolongation	[171]
CS/Quercetin-modified	EDTACitric acid Ascorbic acid	Dipped	Cactus fruits	Improvement of antioxidant and antimicrobial activities	[172]
CS/Whey protein	Hydrochloric acid	Dipped	Ricotta cheese	Texture improvement Sensory characteristics conservation Shelf-life prolongation	[173]
CS/High molecular weight hydroxypropyl methylcellulose	Bergamot essential Acetic acid	Dipped	Grapes	Weight and firmness conservation Respiration rate reduction Antimicrobial activity improvement	[174]
CS/Sodium caseinate	Acetic acid Glycerol	Wrapped Dipped	Carrot Cheese Salami	Inhibition of mesophilic, psychrotrophic, yeast, mold, and bacteria growth	[175]

## Data Availability

Data are contained within the article.

## References

[1] Hosseini S.F., Gómez-Guillén M.C. (2018). A state-of-the-art review on the elaboration of fish gelatin as bioactive packaging: Special emphasis on nanotechnology-based approaches. Trends Food Sci. Technol..

[2] Yai H. (2008). Edible films and coatings: Characteristics and properties. Int. Food Res. J..

[3] Davis G., Song J.H. (2006). Biodegradable packaging based on raw materials from crops and their impact on waste management. Ind. Crops Prod..

[4] Díaz-Montes E., Castro-Muñoz R. (2021). Edible Films and Coatings as Food-Quality Preservers: An Overview. Foods.

[5] Tahir H.E., Xiaobo Z., Mahunu G.K., Arslan M., Abdalhai M., Zhihua L. (2019). Recent developments in gum edible coating applications for fruits and vegetables preservation: A review. Carbohydr. Polym..

[6] Marsh K., Bugusu B. (2007). Food packaging—Roles, materials, and environmental issues: Scientific status summary. J. Food Sci..

[7] Homez-Jara A., Daza L.D., Aguirre D.M., Muñoz J.A., Solanilla J.F., Váquiro H.A. (2018). Characterization of chitosan edible films obtained with various polymer concentrations and drying temperatures. Int. J. Biol. Macromol..

[8] Klemm D., Heublein B., Fink H.P., Bohn A. (2005). Cellulose: Fascinating biopolymer and sustainable raw material. Angew. Chemie Int. Ed..

[9] Das B., Patra S. (2017). Antimicrobials: Meeting the Challenges of Antibiotic Resistance through Nanotechnology.

[10] De Ven A.L.V., MacK A., Dunner K., Ferrari M., Serda R.E. (2012). Preparation, characterization, and cellular associations of silicon logic-embedded vectors. Methods in Enzymology.

[11] Şenel S., McClure S.J. (2004). Potential applications of chitosan in veterinary medicine. Adv. Drug Deliv. Rev..

[12] Thapa B., Narain R., Narain R. (2016). Mechanism, current challenges and new approaches for non viral gene delivery. Polymers and Nanomaterials for Gene Therapy.

[13] Castro-Muñoz R., González-Valdez J., Ahmad M.Z. (2020). High-performance pervaporation chitosan-based membranes: New insights and perspectives. Rev. Chem. Eng..

[14] Rinaudo M. (2006). Chitin and chitosan: Properties and applications. Prog. Polym. Sci..

[15] Kristl J., Šmid-Korbar J., Štruc E., Schara M., Rupprecht H. (1993). Hydrocolloids and gels of chitosan as drug carriers. Int. J. Pharm..

[16] Kong M., Chen X.G., Xing K., Park H.J. (2010). Antimicrobial properties of chitosan and mode of action: A state of the art review. Int. J. Food Microbiol..

[17] Castro Fernández H., Ledea Lozano O. (2010). Determinación de la bioactividad y la resistencia a la compresion de bloques de poliapatita. Quim. Nov..

[18] Bautista-Baños S., Romanazzi G., Aiménez-Aparicio A. (2016). Chitosan in the Preservation of Agritultural Commodities.

[19] Lim S.H., Hudson S.M. (2004). Synthesis and antimicrobial activity of a water-soluble chitosan derivative with a fiber-reactive group. Carbohydr. Res..

[20] Kong M., Chen X.G., Xue Y.P., Liu C.S., Yu L.J., Ji Q.X., Cha D.S., Park H.J. (2008). Preparation and antibacterial activity of chitosan microshperes in a solid dispersing system. Front. Mater. Sci. China.

[21] Castro-Muñoz R., Gonzalez-Melgoza L., Garcia-Depraect O. (2021). Ongoing progress on novel nanocomposite membranes for the separation of heavy metals from contaminated water. Chemosphere.

[22] Xing K., Chen X.G., Liu C.S., Cha D.S., Park H.J. (2009). Oleoyl-chitosan nanoparticles inhibits *Escherichia coli* and *Staphylococcus aureus* by damaging the cell membrane and putative binding to extracellular or intracellular targets. Int. J. Food Microbiol..

[23] Shahidi F., Arachchi J.K.V., Jeon Y.J. (1999). Food applications of chitin and chitosans. Trends Food Sci. Technol..

[24] Chirkov S.N. (2002). The antiviral activity of chitosan (review). Appl. Biochem. Microbiol..

[25] Matica M.A., Aachmann F.L., Tøndervik A., Sletta H., Ostafe V. (2019). Chitosan as a wound dressing starting material: Antimicrobial properties and mode of action. Int. J. Mol. Sci..

[26] Montalvo C., López Malo A., Palou E. (2012). Películas comestibles de proteína: Características, propiedades y aplicaciones. Temas Sel. Ing. Aliment..

[27] Embuscado M.E., Huber K.C. (2009). Edible Films and Coatings for Food Applications.

[28] Guimarães A., Abrunhosa L., Pastrana L.M., Cerqueira M.A. (2018). Edible Films and Coatings as Carriers of Living Microorganisms: A New Strategy Towards Biopreservation and Healthier Foods. Compr. Rev. Food Sci. Food Saf..

[29] Azevedo V.M., Dias M.V., de Siqueira Elias H.H., Fukushima K.L., Silva E.K., de Deus Souza Carneiro J., de Fátima Ferreira Soares N., Borges S.V. (2018). Effect of whey protein isolate films incorporated with montmorillonite and citric acid on the preservation of fresh-cut apples. Food Res. Int..

[30] Rangel-Marrón M., Mani-López E., Palou E., López-Malo A. (2019). Effects of alginate-glycerol-citric acid concentrations on selected physical, mechanical, and barrier properties of papaya puree-based edible films and coatings, as evaluated by response surface methodology. LWT.

[31] Cerqueira M.A., Sousa-Gallagher M.J., Macedo I., Rodriguez-Aguilera R., Souza B.W.S., Teixeira J.A., Vicente A.A. (2010). Use of galactomannan edible coating application and storage temperature for prolonging shelf-life of “Regional” cheese. J. Food Eng..

[32] Nallan Chakravartula S.S., Cevoli C., Balestra F., Fabbri A., Dalla Rosa M. (2019). Evaluation of drying of edible coating on bread using NIR spectroscopy. J. Food Eng..

[33] Tavassoli-Kafrani E., Shekarchizadeh H., Masoudpour-Behabadi M. (2016). Development of edible films and coatings from alginates and carrageenans. Carbohydr. Polym..

[34] Guerrero Legarreta I., Rosmini M.R., Armenta R.E. (2009). Tecnología de Productos de Origen Acuático.

[35] Debeaufort F., Quezada-Gallo J.-A., Voilley A. (1998). Edible films and coatings: Tomorrow’s packagings: A review. Crit. Rev. Food Sci. Nutr..

[36] Falguera V., Quintero J.P., Jiménez A., Muñoz J.A., Ibarz A. (2011). Edible films and coatings: Structures, active functions and trends in their use. Trends Food Sci. Technol..

[37] Salvia-Trujillo L., Soliva-Fortuny R., Rojas-Graü M.A., McClements D.J., Martín-Belloso O. (2017). Edible Nanoemulsions as Carriers of Active Ingredients: A Review. Annu. Rev. Food Sci. Technol..

[38] Park S.-I., Zhao Y. (2004). Incorporation of a high concentration of mineral or vitamin into chitosan-based films. J. Agric. Food Chem..

[39] Liu J., Liu S., Chen Y., Zhang L., Kan J., Jin C. (2017). Physical, mechanical and antioxidant properties of chitosan films grafted with different hydroxybenzoic acids. Food Hydrocoll..

[40] Biale J.B. (1964). Growth, maturation, and senescence in fruits: Recent knowledge on growth regulation and on biological oxidations has been applied to studies with fruits. Science.

[41] Li H., Huneault M.A. (2011). Comparison of sorbitol and glycerol as plasticizers for thermoplastic starch in TPS/PLA blends. J. Appl. Polym. Sci..

[42] Tadros T. (2013). Surfactants. Encyclopedia of Colloid and Interface Science.

[43] Rubilar J.F., Cruz R.M.S., Silva H.D., Vicente A.A., Khmelinskii I., Vieira M.C. (2013). Physico-mechanical properties of chitosan films with carvacrol and grape seed extract. J. Food Eng..

[44] Park S.Y., Marsh K.S., Rhim J.W. (2002). Characteristics of different molecular weight chitosan films affected by the type of organic solvents. J. Food Sci..

[45] Castro-Muñoz R., Agrawal K.V., Coronas J. (2020). Ultrathin permselective membranes: The latent way for efficient gas separation. RSC Adv..

[46] Campos C.A., Gerschenson L.N., Flores S.K. (2011). Development of edible films and coatings with antimicrobial activity. Food Bioprocess Technol..

[47] Wani A.A., Singh P., Langowski H.-C., Singh P., Wani A.A., Langowski H.-C. (2017). Introduction: Food Packaging Materials. Food Packaging Materials: Testing & Quality Assurance.

[48] Kanatt S.R., Muppalla S.R., Chawla S.P., Thakur V.K., Thakur M.K., Kessñer M.R. (2017). Eco-friendly polymers for food packaging. Handbook of Composites from Renewable Materials.

[49] Abraham S., Rajamanick D., Srinivasan B. (2018). Preparation, characterization and cross-linking of chitosan by Microwave Assisted Synthesis. Sci. Int..

[50] Kaya M., Khadem S., Cakmak Y.S., Mujtaba M., Ilk S., Akyuz L., Salaberria A.M., Labidi J., Abdulqadir A.H., Deligöz E. (2018). Antioxidative and antimicrobial edible chitosan films blended with stem, leaf and seed extracts of *Pistacia terebinthus* for active food packaging. RSC Adv..

[51] Yin M., Lin X., Ren T., Li Z., Ren X., Huang T.S. (2018). Cytocompatible quaternized carboxymethyl chitosan/poly(vinyl alcohol) blend film loaded copper for antibacterial application. Int. J. Biol. Macromol..

[52] Beigzadeh Ghelejlu S., Esmaiili M., Almasi H. (2016). Characterization of chitosan-nanoclay bionanocomposite active films containing milk thistle extract. Int. J. Biol. Macromol..

[53] Hafsa J., Smach M.A., Ben Khedher M.R., Charfeddine B., Limem K., Majdoub H., Rouatbi S. (2016). Physical, antioxidant and antimicrobial properties of chitosan films containing *Eucalyptus globulus* essential oil. LWT Food Sci. Technol..

[54] Perdones Á., Chiralt A., Vargas M. (2016). Properties of film-forming dispersions and films based on chitosan containing basil or thyme essential oil. Food Hydrocoll..

[55] Siripatrawan U., Vitchayakitti W. (2016). Improving functional properties of chitosan films as active food packaging by incorporating with propolis. Food Hydrocoll..

[56] Costa M.J., Cerqueira M.A., Ruiz H.A., Fougnies C., Richel A., Vicente A.A., Teixeira J.A., Aguedo M. (2015). Use of wheat bran arabinoxylans in chitosan-based films: Effect on physicochemical properties. Ind. Crops Prod..

[57] Fernández-Pan I., Maté J.I., Gardrat C., Coma V. (2015). Effect of chitosan molecular weight on the antimicrobial activity and release rate of carvacrol-enriched films. Food Hydrocoll..

[58] Kurek M., Guinault A., Voilley A., Galić K., Debeaufort F. (2014). Effect of relative humidity on carvacrol release and permeation properties of chitosan based films and coatings. Food Chem..

[59] Sun X., Wang Z., Kadouh H., Zhou K. (2014). The antimicrobial, mechanical, physical and structural properties of chitosan-gallic acid films. LWT Food Sci. Technol..

[60] Ulbin-Figlewicz N., Zimoch-Korzycka A., Jarmoluk A. (2014). Antibacterial Activity and Physical Properties of Edible Chitosan Films Exposed to Low-pressure Plasma. Food Bioprocess Technol..

[61] Peng Y., Li Y. (2014). Combined effects of two kinds of essential oils on physical, mechanical and structural properties of chitosan films. Food Hydrocoll..

[62] Wang L., Dong Y., Men H., Tong J., Zhou J. (2013). Preparation and characterization of active films based on chitosan incorporated tea polyphenols. Food Hydrocoll..

[63] Zhang Y., Peng J., Wang J. (2013). Preparation and characterization of chitosan edible film. Appl. Mech. Mater..

[64] Ruiz-Navajas Y., Viuda-Martos M., Sendra E., Perez-Alvarez J.A., Fernández-López J. (2013). In vitro antibacterial and antioxidant properties of chitosan edible films incorporated with *Thymus moroderi* or *Thymus piperella* essential oils. Food Control.

[65] Kanatt S.R., Rao M.S., Chawla S.P., Sharma A. (2012). Active chitosan-polyvinyl alcohol films with natural extracts. Food Hydrocoll..

[66] Pereda M., Amica G., Marcovich N.E. (2012). Development and characterization of edible chitosan/olive oil emulsion films. Carbohydr. Polym..

[67] Avila-Sosa R., Palou E., Jiménez Munguía M.T., Nevárez-Moorillón G.V., Navarro Cruz A.R., López-Malo A. (2012). Antifungal activity by vapor contact of essential oils added to amaranth, chitosan, or starch edible films. Int. J. Food Microbiol..

[68] Cerqueira M.A., Souza B.W.S., Teixeira J.A., Vicente A.A. (2012). Effects of interactions between the constituents of chitosan-edible films on their physical properties. Food Bioprocess Technol..

[69] Cissé M., Montet D., Loiseau G., Ducamp-Collin M.N. (2012). Influence of the concentrations of chitosan and glycerol on edible film properties showed by response surface methodology. J. Polym. Environ..

[70] Martins J.T., Cerqueira M.A., Vicente A.A. (2012). Influence of α-tocopherol on physicochemical properties of chitosan-based films. Food Hydrocoll..

[71] Moradi M., Tajik H., Razavi Rohani S.M., Oromiehie A.R., Malekinejad H., Aliakbarlu J., Hadian M. (2012). Characterization of antioxidant chitosan film incorporated with *Zataria multiflora* Boiss essential oil and grape seed extract. LWT Food Sci. Technol..

[72] Bonilla J., Vargas M., Atarés L., Chiralt A. (2011). Physical properties of chitosan-basil essential oil edible films as affected by oil content and homogenization conditions. Procedia Food Sci..

[73] Bonilla J., Atarés L., Vargas M., Chiralt A. (2012). Effect of essential oils and homogenization conditions on properties of chitosan-based films. Food Hydrocoll..

[74] Avila-Sosa R., Hernández-Zamoran E., López-Mendoza I., Palou E., Munguía M.T.J., Nevárez-Moorillón G.V., López-Malo A. (2010). Fungal inactivation by Mexican oregano (*Lippia berlandieri* Schauer) essential oil added to amaranth, chitosan, or starch edible films. J. Food Sci..

[75] Sánchez-González L., Cháfer M., Chiralt A., González-Martínez C. (2010). Physical properties of edible chitosan films containing bergamot essential oil and their inhibitory action on *Penicillium italicum*. Carbohydr. Polym..

[76] Souza B.W.S., Cerqueira M.A., Martins J.T., Casariego A., Teixeira J.A., Vicente A.A. (2010). Influence of electric fields on the structure of chitosan edible coatings. Food Hydrocoll..

[77] Ojagh S.M., Rezaei M., Razavi S.H., Hosseini S.M.H. (2010). Development and evaluation of a novel biodegradable film made from chitosan and cinnamon essential oil with low affinity toward water. Food Chem..

[78] Martínez-Camacho A.P., Cortez-Rocha M.O., Ezquerra-Brauer J.M., Graciano-Verdugo A.Z., Rodriguez-Félix F., Castillo-Ortega M.M., Yépiz-Gómez M.S., Plascencia-Jatomea M. (2010). Chitosan composite films: Thermal, structural, mechanical and antifungal properties. Carbohydr. Polym..

[79] Zemljič L.F., Ulrih N.P., Sterniša M., Možina S.S., Plohl O., Glaser T.K., Valh J.V. (2019). Pullulan-chitosan coatings onto polyethylene foils for the development of active packaging material. Cellul. Chem. Technol..

[80] Zhang W., Xiao H., Qian L. (2014). Beeswax-chitosan emulsion coated paper with enhanced water vapor barrier efficiency. Appl. Surf. Sci..

[81] Kurek M., Galus S., Debeaufort F. (2014). Surface, mechanical and barrier properties of bio-based composite films based on chitosan and whey protein. Food Packag. Shelf Life.

[82] Velickova E., Winkelhausen E., Kuzmanova S., Alves V.D., Moldão-Martins M. (2013). Impact of chitosan-beeswax edible coatings on the quality of fresh strawberries (*Fragaria ananassa* cv Camarosa) under commercial storage conditions. LWT Food Sci. Technol..

[83] Bonilla J., Atarés L., Vargas M., Chiralt A. (2013). Properties of wheat starch film-forming dispersions and films as affected by chitosan addition. J. Food Eng..

[84] Kurek M., Ščetar M., Voilley A., Galić K., Debeaufort F. (2012). Barrier properties of chitosan coated polyethylene. J. Memb. Sci..

[85] Pereda M., Ponce A.G., Marcovich N.E., Ruseckaite R.A., Martucci J.F. (2011). Chitosan-gelatin composites and bi-layer films with potential antimicrobial activity. Food Hydrocoll..

[86] Fardioui M., Meftah Kadmiri I., Qaiss A.e.K., Bouhfid R. (2018). Bio-active nanocomposite films based on nanocrystalline cellulose reinforced styrylquinoxalin-grafted-chitosan: Antibacterial and mechanical properties. Int. J. Biol. Macromol..

[87] Jahed E., Khaledabad M.A., Almasi H., Hasanzadeh R. (2017). Physicochemical properties of *Carum copticum* essential oil loaded chitosan films containing organic nanoreinforcements. Carbohydr. Polym..

[88] Talón E., Trifkovic K.T., Nedovic V.A., Bugarski B.M., Vargas M., Chiralt A., González-Martínez C. (2017). Antioxidant edible films based on chitosan and starch containing polyphenols from thyme extracts. Carbohydr. Polym..

[89] Arifin B., Sugita P., Masyudi D.E. (2016). Chitosan and lauric acid addition to corn starch-film based effect: Physical properties and antimicrobial activity study. Int. J. Chem. Sci..

[90] Benbettaïeb N., Chambin O., Karbowiak T., Debeaufort F. (2016). Release behavior of quercetin from chitosan-fish gelatin edible films influenced by electron beam irradiation. Food Control.

[91] Bonilla J., Sobral P.J.A. (2016). Investigation of the physicochemical, antimicrobial and antioxidant properties of gelatin-chitosan edible film mixed with plant ethanolic extracts. Food Biosci..

[92] Bof M.J., Bordagaray V.C., Locaso D.E., García M.A. (2015). Chitosan molecular weight effect on starch-composite film properties. Food Hydrocoll..

[93] Alvarado S., Sandoval G., Palos I., Tellez S., Aguirre-Loredo Y., Velazquez G. (2015). The effect of relative humidity on tensile strength and water vapor permeability in chitosan, fish gelatin and transglutaminase edible films. Food Sci. Technol..

[94] Antoniou J., Liu F., Majeed H., Zhong F. (2015). Characterization of tara gum edible films incorporated with bulk chitosan and chitosan nanoparticles: A comparative study. Food Hydrocoll..

[95] Cheng S.Y., Wang B.J., Weng Y.M. (2015). Antioxidant and antimicrobial edible zein/chitosan composite films fabricated by incorporation of phenolic compounds and dicarboxylic acids. LWT Food Sci. Technol..

[96] Santacruz S., Rivadeneira C., Castro M. (2015). Edible films based on starch and chitosan. Effect of starch source andconcentration, plasticizer, surfactant’s hydrophobic tail andmechanical treatment. Food Hydrocoll..

[97] Ashori A., Bahrami R. (2014). Modification of physico-mechanical properties of chitosan-tapioca starch blend films using nano graphene. Polym. Plast. Technol. Eng..

[98] Escamilla-García M., Calderón-Domínguez G., Chanona-Pérez J.J., Farrera-Rebollo R.R., Andraca-Adame J.A., Arzate-Vázquez I., Mendez-Mendez J.V., Moreno-Ruiz L.A. (2013). Physical and structural characterisation of zein and chitosan edible films using nanotechnology tools. Int. J. Biol. Macromol..

[99] Hosseini S.F., Rezaei M., Zandi M., Ghavi F.F. (2013). Preparation and functional properties of fish gelatin-chitosan blend edible films. Food Chem..

[100] Pastor C., Sánchez-González L., Chiralt A., Cháfer M., González-Martínez C. (2013). Physical and antioxidant properties of chitosan and methylcellulose based films containing resveratrol. Food Hydrocoll..

[101] Bhattacharya T., Bandyopadhyay N.R., Ray D., Bhattacharyya D.K., Das K.P. (2013). Preparation and characterizations of soya protein isolate films coated with palm stearin and modified with antimicrobial agent chitosan. Int. J. Adv. Res. Technol..

[102] Silva-Weiss A., Bifani V., Ihl M., Sobral P.J.A., Gómez-Guillén M.C. (2013). Structural properties of films and rheology of film-forming solutions based on chitosan and chitosan-starch blend enriched with murta leaf extract. Food Hydrocoll..

[103] Sionkowska A., Płanecka A. (2013). Surface properties of thin films based on the mixtures of chitosan and silk fibroin. J. Mol. Liq..

[104] Valenzuela C., Abugoch L., Tapia C. (2013). Quinoa protein-chitosan-sunflower oil edible film: Mechanical, barrier and structural properties. LWT Food Sci. Technol..

[105] Wu J., Zhong F., Li Y., Shoemaker C.F., Xia W. (2013). Preparation and characterization of pullulan-chitosan and pullulan-carboxymethyl chitosan blended films. Food Hydrocoll..

[106] Arzate-Vázquez I., Chanona-Pérez J.J., Calderón-Domínguez G., Terres-Rojas E., Garibay-Febles V., Martínez-Rivas A., Gutiérrez-López G.F. (2012). Microstructural characterization of chitosan and alginate films by microscopy techniques and texture image analysis. Carbohydr. Polym..

[107] Mehdizadeh T., Tajik H., Razavi Rohani S.M., Oromiehie A.R. (2012). Antibacterial, antioxidant and optical properties of edible starch-chitosan composite film containing Thymus kotschyanus essential oil. Vet. Res. Forum.

[108] De Moura M.R., Lorevice M.V., Mattoso L.H.C., Zucolotto V. (2011). Highly stable, edible cellulose films incorporating chitosan nanoparticles. J. Food Sci..

[109] Abugoch L.E., Tapia C., Villamán M.C., Yazdani-Pedram M., Díaz-Dosque M. (2011). Characterization of quinoa protein-chitosan blend edible films. Food Hydrocoll..

[110] Bourbon A.I., Pinheiro A.C., Cerqueira M.A., Rocha C.M.R., Avides M.C., Quintas M.A.C., Vicente A.A. (2011). Physico-chemical characterization of chitosan-based edible films incorporating bioactive compounds of different molecular weight. J. Food Eng..

[111] Azeredo H.M.C., Mattoso L.H.C., Avena-Bustillos R.J., Filho G.C., Munford M.L., Wood D., McHugh T.H. (2010). Nanocellulose reinforced chitosan composite films as affected by nanofiller loading and plasticizer content. J. Food Sci..

[112] Tripathi S., Mehrotra G.K., Dutta P.K. (2010). Preparation and physicochemical evaluation of chitosan/poly(vinyl alcohol)/pectin ternary film for food-packaging applications. Carbohydr. Polym..

[113] Zhu L.Y., Lin D.Q., Yao S.J. (2010). Biodegradation of polyelectrolyte complex films composed of chitosan and sodium cellulose sulfate as the controllable release carrier. Carbohydr. Polym..

[114] Farahnaky A., Saberi B., Majzoobi M. (2013). Effect of glycerol on physical and mechanical properties of wheat starch edible films. J. Texture Stud..

[115] Kemp I.C. (2007). Humidity effects in solids drying processes. Meas. Control.

[116] Soazo M., Rubiolo A.C., Verdini R.A. (2011). Effect of drying temperature and beeswax content on physical properties of whey protein emulsion films. Food Hydrocoll..

[117] Scio E., Mendes R.F., Motta E.V.S., Bellozi P.M.Q., Aragão D.M.O., Mello J., Fabri R.L., Moreira J.R., de Assis I.V.L., Bouz M.L.M., Rao V. (2012). Antimicrobial and Antioxidant Activities of Some Plant Extracts. Phytochemicals as Nutraceuticals—Global Approaches to Their Role in Nutrition and Health.

[118] El-Naggar M.N., Abdulla G., El-Shourbagy G.A., El-Badawi A.A., El Sohaimy S.A. (2017). Antimicrobial and antioxidant activities of some plant extracts. Zagazig J. Agric. Res..

[119] Castro-Muñoz R., Díaz-Montes E., Cassano A., Gontarek E. (2020). Membrane separation processes for the extraction and purification of steviol glycosides: An overview. Crit. Rev. Food Sci. Nutr..

[120] Díaz-Montes E., Castro-Muñoz R. (2019). Metabolites recovery from fermentation broths via pressure-driven membrane processes. Asia-Pac. J. Chem. Eng..

[121] Safari M., Ahmady-Asbchin S. (2019). Evaluation of antioxidant and antibacterial activities of methanolic extract of medlar (*Mespilus germanica* L.) leaves. Biotechnol. Biotechnol. Equip..

[122] Park C.H., Yeo H.J., Baskar T.B., Park Y.E., Park J.S., Lee S.Y., Park S.U. (2019). In vitro antioxidant and antimicrobial properties of flower, leaf, and stem extracts of Korean mint. Antioxidants.

[123] Castro-Muñoz R., Buera-González J., de la Iglesia Ó., Galiano F., Fíla V., Malankowska M., Rubio C., Figoli A., Téllez C., Coronas J. (2019). Towards the dehydration of ethanol using pervaporation cross-linked poly(vinyl alcohol)/graphene oxide membranes. J. Memb. Sci..

[124] Siripatrawan U., Vitchayakitti W., Sanguandeekul R. (2013). Antioxidant and antimicrobial properties of Thai propolis extracted using ethanol aqueous solution. Int. J. Food Sci. Technol..

[125] Bhavaniramya S., Vishnupriya S., Al-Aboody M.S., Vijayakumar R., Baskaran D. (2019). Role of essential oils in food safety: Antimicrobial and antioxidant applications. Grain Oil Sci. Technol..

[126] Elansary H.O., Abdelgaleil S.A.M., Mahmoud E.A., Yessoufou K., Elhindi K., El-Hendawy S. (2018). Effective antioxidant, antimicrobial and anticancer activities of essential oils of horticultural aromatic crops in northern Egypt. BMC Complement. Altern. Med..

[127] Zhang X., Guo Y., Guo L., Jiang H., Ji Q. (2018). In vitro evaluation of antioxidant and antimicrobial activities of *Melaleuca alternifolia* essential oil. Biomed Res. Int..

[128] Kokina M., Salevic A., Kaluševic A., Levic S., Pantic M., Pljevljakušic D., Šavikin K., Shamtsyan M., Nikšic M., Nedovic V. (2019). Characterization, antioxidant and antibacterial activity of essential oils and their encapsulation into biodegradable material followed by freeze drying. Food Technol. Biotechnol..

[129] Kurti F., Giorgi A., Beretta G., Mustafa B., Gelmini F., Testa C., Angioletti S., Giupponi L., Zilio E., Pentimalli D. (2019). Chemical composition, antioxidant and antimicrobial activities of essential oils of different Pinus species from Kosovo. J. Essent. Oil Res..

[130] Ebrahimzadeh M.A., Nabavi S.F., Nabavi S.M. (2009). Antioxidant activities of methanol extract of *Sambucus ebulus* L. flower. Pakistan J. Biol. Sci..

[131] Nabavi S.M., Ebrahimzadeh M.A., Nabavi S.F., Jafari M. (2008). Free radical scavenging activity and antioxidant capacity of *Eryngium caucasicum* Trautv and *Froripia subpinnata*. Pharmacologyonline.

[132] Shellhammer T.H., Krochta J.M. (1997). Whey protein emulsion film performance as affected by lipid type and amount. J. Food Sci..

[133] Zhang W., Xiao H., Qian L. (2014). Enhanced water vapour barrier and grease resistance of paper bilayer-coated with chitosan and beeswax. Carbohydr. Polym..

[134] Kong F., Singh R.P., Subramaniam P. (2016). Chemical deterioration and physical instability of foods and beverages. The Stability and Shelf Life of Food.

[135] Ruiz-Cruz S., Valenzuela-López C.C., Chaparro-Hernández S., Ornelas-Paz J.D.J., Del Toro-Sánchez C.L., Márquez-Ríos E., López-Mata M.A., Ocaño-Higuera V.M., Valdez-Hurtado S. (2019). Effect of chitosan-tomato plant extract edible coating on the quality and shelf life of chicken fillets during refrigerated storage. Food Sci. Technol..

[136] Brown S.R.B., Kozak S.M., D’Amico D.J. (2018). Applications of Edible Coatings Formulated with Antimicrobials Inhibit Listeria monocytogenes Growth on Queso Fresco. Front. Sustain. Food Syst..

[137] Apriyanti D., Rokhati N., Mawarni N., Khoiriyah Z., Istirokhatun T. (2018). Edible coating from green tea extract and chitosan to preserve strawberry (*Fragaria vesca* L.). MATEC Web Conf..

[138] Karimnezhad F., Razavilar V., Anvar A.A., Eskandari S. (2017). Study the antimicrobial effects of chitosan-based edible film containing the Trachyspermum ammi essential oil on shelf-life of chicken meat. Microbiol. Res. (Pavia).

[139] Abriana A., Laga S. (2016). Application of Edible Coating from Chitosan Skin Shrimp (*Paneus monodon*) to Apple (*Malus sylvestris*) Minimum Processed. J. Food Stud..

[140] Leceta I., Molinaro S., Guerrero P., Kerry J.P., De la Caba K. (2015). Quality attributes of map packaged ready-to-eat baby carrots by using chitosan-based coatings. Postharvest Biol. Technol..

[141] Ruiz-Navajas Y., Viuda-Martos M., Barber X., Sendra E., Perez-Alvarez J.A., Fernández-López J. (2015). Effect of chitosan edible films added with *Thymus moroderi* and *Thymus piperella* essential oil on shelf-life of cooked cured ham. J. Food Sci. Technol..

[142] Petriccione M., Pasquariello M.S., Mastrobuoni F., Zampella L., Di Patre D., Scortichini M. (2015). Influence of a chitosan coating on the quality and nutraceutical traits of loquat fruit during postharvest life. Sci. Hortic. (Amst.).

[143] Liu K., Yuan C., Chen Y., Li H., Liu J. (2014). Combined effects of ascorbic acid and chitosan on the quality maintenance and shelf life of plums. Sci. Hortic. (Amst.).

[144] Aşik E., Candoğan K. (2014). Effects of chitosan coatings incorporated with garlic oill on quality characteristics of shrimp. J. Food Qual..

[145] Günlü A., Koyun E. (2013). Effects of vacuum packaging and wrapping with chitosan-based edible film on the extension of the shelf life of sea bass (*Dicentrarchus labrax*) fillets in cold storage (4 °C). Food Bioprocess Technol..

[146] Hong K., Xie J., Zhang L., Sun D., Gong D. (2012). Effects of chitosan coating on postharvest life and quality of guava (*Psidium guajava* L.) fruit during cold storage. Sci. Hortic. (Amst.).

[147] Mohan C.O., Ravishankar C.N., Lalitha K.V., Srinivasa Gopal T.K. (2012). Effect of chitosan edible coating on the quality of double filleted Indian oil sardine (*Sardinella longiceps*) during chilled storage. Food Hydrocoll..

[148] Perdones A., Sánchez-González L., Chiralt A., Vargas M. (2012). Effect of chitosan-lemon essential oil coatings on storage-keeping quality of strawberry. Postharvest Biol. Technol..

[149] Zaki N.H.M., Som H.Z.M., Haiyee Z.A. (2012). Application of palm stearin-chitosan edible coating on star fruits (*Averrhoa carambola* L.). Malays. J. Anal. Sci..

[150] Moradi M., Tajik H., Razavi Rohani S.M., Oromiehie A.R. (2011). Effectiveness of *Zataria multiflora* Boiss essential oil and grape seed extract impregnated chitosan film on ready-to-eat mortadella-type sausages during refrigerated storage. J. Sci. Food Agric..

[151] Moreira M.d.R., Roura S.I., Ponce A. (2011). Effectiveness of chitosan edible coatings to improve microbiological and sensory quality of fresh cut broccoli. LWT Food Sci. Technol..

[152] Garrido Assis O.B., de Britto D. (2011). Evaluation of the antifungal properties of chitosan coating on cut apples using a non-invasive image analysis technique. Polym. Int..

[153] Vargas M., Albors A., Chiralt A. (2011). Application of chitosan-sunflower oil edible films to pork meat hamburgers. Procedia Food Sci..

[154] Jiang Z., Neetoo H., Chen H. (2011). Control of Listeria Monocytogenes on cold-smoked salmon using chitosan-based antimicrobial coatings and films. J. Food Sci..

[155] Duan J., Cherian G., Zhao Y. (2010). Quality enhancement in fresh and frozen lingcod (*Ophiodon elongates*) fillets by employment of fish oil incorporated chitosan coatings. Food Chem..

[156] Fajardo P., Martins J.T., Fuciños C., Pastrana L., Teixeira J.A., Vicente A.A. (2010). Evaluation of a chitosan-based edible film as carrier of natamycin to improve the storability of Saloio cheese. J. Food Eng..

[157] Nisha V., Monisha C., Ragunathan R., Johney J. (2016). Use of chitosan as edible coating on fruits and in micro biological activity-an ecofriendly approach. Int. J. Pharm. Sci. Invent..

[158] Vu K.D., Hollingsworth R.G., Leroux E., Salmieri S., Lacroix M. (2011). Development of edible bioactive coating based on modified chitosan for increasing the shelf life of strawberries. Food Res. Int..

[159] Xiao Z., Luo Y., Luo Y., Wang Q. (2011). Combined effects of sodium chlorite dip treatment and chitosan coatings on the quality of fresh-cut d’Anjou pears. Postharvest Biol. Technol..

[160] Jovanovic G.D., Klaus A.S., Niksic M.P. (2016). Antimicrobial activity of chitosan films with essential oils against Listeria monocytogenes on cabbage. Rev. Argent. Microbiol..

[161] Cardoso G.P., Dutra M.P., Fontes P.R., Ramos A.D.L.S., de Miranda Gomide L.A., Ramos E.M. (2016). Selection of a chitosan gelatin-based edible coating for color preservation of beef in retail display. Meat Sci..

[162] Nowzari F., Shábanpour B., Ojagh S.M. (2013). Comparison of chitosan-gelatin composite and bilayer coating and film effect on the quality of refrigerated rainbow trout. Food Chem..

[163] Gómez-Estaca J., López de Lacey A., López-Caballero M.E., Gómez-Guillén M.C., Montero P. (2010). Biodegradable gelatin-chitosan films incorporated with essential oils as antimicrobial agents for fish preservation. Food Microbiol..

[164] Abugoch L., Tapia C., Plasencia D., Pastor A., Castro-Mandujano O., López L., Escalona V.H. (2016). Shelf-life of fresh blueberries coated with quinoa protein/chitosan/sunflower oil edible film. J. Sci. Food Agric..

[165] Yusof N.M., Jai J., Hamzah F., Pinijsuwan S. (2018). Evaluation of the Effect of *Curcuma longa* L. Essential Oil in Chitosan-starch Edible Coating. IOP Conf. Ser. Mater. Sci. Eng..

[166] Azevedo A.N., Buarque P.R., Cruz E.M.O., Blank A.F., Alves P.B., Nunes M.L., de Aquino Santana L.C.L. (2014). Response surface methodology for optimisation of edible chitosan coating formulations incorporating essential oil against several foodborne pathogenic bacteria. Food Control.

[167] Mei J., Yuan Y., Wu Y., Li Y. (2013). Characterization of edible starch-chitosan film and its application in the storage of Mongolian cheese. Int. J. Biol. Macromol..

[168] Lin M.G., Lasekan O., Saari N., Khairunniza-Bejo S. (2018). Effect of chitosan and carrageenan-based edible coatings on post-harvested longan (*Dimocarpus longan*) fruits. CYTA J. Food.

[169] Poverenov E., Danino S., Horev B., Granit R., Vinokur Y., Rodov V. (2014). Layer-by-layer electrostatic deposition of edible coating on fresh cut melon model: Anticipated and unexpected effects of alginate-chitosan combination. Food Bioprocess Technol..

[170] Arnon H., Zaitsev Y., Porat R., Poverenov E. (2014). Effects of carboxymethyl cellulose and chitosan bilayer edible coating on postharvest quality of citrus fruit. Postharvest Biol. Technol..

[171] Huang J., Chen Q., Qiu M., Li S. (2012). Chitosan-based edible coatings for quality preservation of postharvest whiteleg shrimp (*Litopenaeus vannamei*). J. Food Sci..

[172] Torres E., Marín V., Aburto J., Beltrán H.I., Shirai K., Villanueva S., Sandoval G. (2012). Enzymatic modification of chitosan with quercetin and its application as antioxidant edible films. Appl. Biochem. Microbiol..

[173] Di Pierro P., Sorrentino A., Mariniello L., Giosafatto C.V.L., Porta R. (2011). Chitosan/whey protein film as active coating to extend Ricotta cheese shelf-life. LWT Food Sci. Technol..

[174] Sanchez-Gonzalez L., Pastor C., Vargas M., Chiralt A., Gonzalez-Martinez C., Chafer M. (2011). Effect of hydroxypropylmethylcellulose and chitosan coatings with and without bergamot essential oil on quality and safety of cold-stored grapes. Postharvest Biol. Technol..

[175] Moreira M.d.R., Pereda M., Marcovich N.E., Roura S.I. (2011). Antimicrobial effectiveness of bioactive packaging materials from edible chitosan and casein polymers: Assessment on carrot, cheese, and salami. J. Food Sci..

[176] Roudaut G., Debeaufort F. (2010). Moisture Loss, Gain and Migration in Foods and Its Impact on Food Quality.

[177] Remize F. (2016). Spore-Forming Bacteria. The Microbiological Quality of Food.

[178] Keshri N., Weltzien C., Mahajan P.V. (2019). Sensors for measurement of respiratory gases in fresh produce packaging and storage. Ref. Modul. Food Sci..

[179] Lampila L.E., McMillin K.W., Kerry J.P. (2012). Major microbial hazards associated with packaged seafood. Advances in Meat, Poultry and Seafood Packaging.

[180] Reddy M.V.B., Angers P., Gosselin A., Arul J. (1998). Characterization and use of essential oil from *Thymus vulgaris* against *Botrytis cinerea* and *Rhizopus stolonifer* in strawberry fruits. Phytochemistry.

[181] Fratini F., Cilia G., Turchi B., Felicioli A. (2016). Beeswax: A minireview of its antimicrobial activity and its application in medicine. Asian Pac. J. Trop. Med..

[182] Özyurt G., Kuley E., Özkütük S., Özogul F. (2009). Sensory, microbiological and chemical assessment of the freshness of red mullet (*Mullus barbatus*) and goldband goatfish (*Upeneus moluccensis*) during storage in ice. Food Chem..

[183] Rostamzad H., Shabanpour B., Shabani A., Shahiri H. (2011). Enhancement of the storage quality of frozen Persian sturgeon fillets by using of ascorbic acid. Int. Food Res. J..

[184] Beuchat L.R., Komitopoulou E., Beckers H., Betts R.P., Bourdichon F., Fanning S., Joosten H.M., Ter Kuile B.H. (2013). Low-Water Activity Foods: Increased Concern as Vehicles of Foodborne Pathogens. J. Food Prot..

[185] Bakshia P.S., Selvakumar D., Kadirvelu K., Kumar N.S. (2020). Chitosan as an environment friendly biomaterial—A review on recent modifications and applications. Int. J. Biol. Macromol..

[186] Pérez-Guzmán C.J., Castro-Muñoz R. (2020). A review of zein as a potential biopolymer for tissue engineering and nanotechnological applications. Processes.

